# Personalized medicine, digital technology and trust: a Kantian account

**DOI:** 10.1007/s11019-020-09974-z

**Published:** 2020-09-04

**Authors:** Bjørn K. Myskja, Kristin S. Steinsbekk

**Affiliations:** 1 Department of Philosophy and Religious Studies, Norwegian University of Science and Technology – NTNU, Trondheim, Norway; 2 Department of Biomedical Laboratory Science, Norwegian University of Science and Technology – NTNU, Trondheim, Norway

**Keywords:** Genomics, Precision medicine, Reflexive trust, Autonomy, Empowerment

## Abstract

Trust relations in the health services have changed from asymmetrical paternalism to symmetrical autonomy-based participation, according to a common account. The promises of personalized medicine emphasizing empowerment of the individual through active participation in managing her health, disease and well-being, is characteristic of symmetrical trust. In the influential Kantian account of autonomy, active participation in management of own health is not only an opportunity, but an obligation. Personalized medicine is made possible by the digitalization of medicine with an ensuing increased tailoring of diagnostics, treatment and prevention to the individual. The ideal is to increase wellness by minimizing the layer of interpretation and translation between relevant health information and the patient or user. Arguably, this opens for a new level of autonomy through increased participation in treatment and prevention, and by that, increased empowerment of the individual. However, the empirical realities reveal a more complicated landscape disturbed by information ‘noise’ and involving a number of complementary areas of expertise and technologies, hiding the source and logic of data interpretation. This has lead to calls for a return to a mild form of paternalism, allowing expertise coaching of patients and even withholding information, with patients escaping responsibility through blind or lazy trust. This is morally unacceptable, according to Kant’s ideal of enlightenment, as we have a duty to take responsibility by trusting others reflexively, even as patients. Realizing the promises of personalized medicine requires a system of institutional controls of information and diagnostics, accessible for non-specialists, supported by medical expertise that can function as the accountable gate-keeper taking moral responsibility required for an active, reflexive trust.

## Introduction

Tomorrow’s personalized medicine (PM) is characterized by the turn towards digital technologies expressed in the possibility of producing a vast amount of data points, the increased access to such data points, increased potential of interpretation based on these and increased reliability of findings with an ensuing increased tailoring of diagnostics, treatment and prevention (e.g. Flores et al. 2013; Steinhubl 2019; Abe 2016; Mahoney and Asch 2019; NHS 2018). The vision is to increase precision and minimize the layer of interpretation and translation between relevant health information and the patient or user. Arguably, this opens the space for a new level of autonomy within medicine and health care, characterized by increased participation in treatment and prevention, and increased empowerment of the individual. This is in keeping with the Kantian ideal of autonomy as expressed in his influential account of enlightenment, with reduced expert power and reduced paternalism of the kind associated with the traditional doctor-patient relationship. The turn towards personalized medicine, if realized, will also change the dynamics of trust essential to coping with health-related issues, demanding an active, reflexive approach to trust from the users of health services.

Taking a closer look at the empirical realities of these promises reveals a murkier landscape. It is true that the new high throughput (‘-omics’) technologies, by producing vast amounts of data from one individual, provide a better, more detailed picture. At the same time, this picture is more complex and—at least in a transition phase—disturbed by information ‘noise’. The amount of data produced for example in genomics is increasing rapidly and is scattered throughout the internet, in journals and digital repositories, without any common standard for interpretation. To be useful in general, and in the context of health and well-being in particular, data needs to be interpreted. PM is an interdisciplinary endeavour, and relies on a number of complementary areas of expertise. This situation may create new opportunities but also result in novel kinds of mistakes, including excessive use of the tools, with increased tendencies towards overdiagnosis (Vogt et al. 2019). Digitalization involves new kinds of expertise, but many of the expert tasks within medicine and health care are, in addition, replaced by technology through high throughput analysis, automation and artificial intelligence. This reduces the risk of human error, increasing reliability and apparently improving accessibility for non-experts. However, the source and logic of interpretation becomes more hidden by the layers of required technologies and algorithms.

Recently, the field itself has problematized the ideals of empowerment, replacing the ‘personalized’ with ‘precision’ or ‘stratified’ and downplaying the empowerment rhetoric. With this comes a reintroduction of the paternalist ideal and a focus on individuals’ responsibilities toward their social subgroups (Juengst et al. 2016). We argue that this rhetorical shift away from ‘personalized’ and ‘empowerment’ is expressive of a problematic weakening of patient autonomy that undermines the moral responsibilities of patients as well as of health workers. This is evident when taking a closer look at the ethics of trust in this context, as understood within a Kantian framework.

Our main issue is not only how these technological changes will affect trust, but how *should* patient or user trust in health services and medical expertise be affected? Assuming that agents have the freedom to choose whether to trust or not, it follows that trust is subject to moral requirements. Even if there is a renewed commitment to “professional gatekeeping” in PM (Juengst et al. 2016), the technology will open novel rooms for shared decision making and increased opportunities for patients to take control of their situation (taking matters into their own hands). Patients must decide whether and how to trust the many sources of information, interpretation and knowledge and the experts involved—human and non-human alike. When we talk about ‘non-human experts’, we do not mean that artificial intelligence may be experts in the same sense as human beings. Still, they may replace human expertise in many instances, creating novel problems of trust, trustworthiness and accountability (for a discussion of this problem, see Coeckelbergh 2012). The PM user has to trust several information and decision sources, thus distributing trust. Thus, even if the user is more dependent upon expert systems, she also has to make more decisions concerning who or what to trust. Paradoxically, the space for self-reliance and own responsibility is increasing.

Admittedly, many people in need of health services will be vulnerable, also under future PM conditions, as many will lack the capacities necessary for making these decisions concerning trust and will prefer to leave it to the experts, in what is often called blind trust. This might be even more challenging for the use of PM for wellness and disease prevention. These individuals must comply with and act on advice in order to maintain health, although they have no symptoms and are by all accounts healthy (Horne 2017). Regardless of health status, people will still need help to interpret information and choose how to act on the interpreted data—perhaps even more than before. Some will prefer advice from an expert with knowledge and experience. Others will still want directive guidance and not be burdened with the added responsibilities of self-determination in difficult situations. Recently, a study documented that central stakeholders suggest a shift to a new, moderate paternalism as an answer to the information challenges posed by digitalized medicine (Juengst et al. 2016). We will argue that this return of the expert regime is an inadequate answer to the challenges of increased information, and is morally unacceptable. The basis for trust is already altered, due to the primacy of autonomy in contemporary medicine. PM’s intrinsic distribution of responsibilities and use of technology create further obstacles for this proposed return to paternalism.

Based on an analysis of Kant’s concepts of enlightenment, trust and autonomy, we will argue that a return to a blind or, in Kantian terms, ‘lazy’ trust typical of traditional medicine is untenable. We have a duty to exercise active, reflexive trust, based on active access to information as bases for autonomous decisions. Health authorities have a complementary duty to enable reflexivity. This may require a rethinking of the medical gatekeeper role in light of current knowledge of future PM developments. We use a Kantian approach because this is arguably the most influential account of autonomy as the core value in ethics, and trust and trustworthiness is at the core of Kantian moral philosophy. His discussion is highly relevant for the contemporary challenges concerning the relationship between medical expertise and patients. We do not intend to explore what Kant himself would have thought of these challenges, but rather to discuss how to handle the normative challenges of PM based in a general Kantian approach to autonomy and trust.

## PM and genomic medicine—a new approach to diagnostics and treatment

Genomics, the technology that enables us to decode individual genomes, is an essential component of PM (e.g. Burke and Psaty 2007), together with high capacity analytical technologies and wearable sensors enabling longitudinal ‘multi-modal’ monitoring of individuals (Steinhubl 2019; Ozdemir et al. 2009; Schüssler-Fiorenza et al. 2019; Chen and Snyder 2013). Some have criticized the concept of PM (see e.g. Feiler et al. 2017; Coote and Joyner 2015; Vogt et al. 2016; Muse and Topol 2019; Maughan 2017; Vogt et al. 2019), and one could claim that there is a continuum from traditional medicine to genomic medicine, understood as the production and use of personal genomic information based on digital technologies, which will increasingly be the standard within many healthcare specialities for diagnostic, prognostic and therapeutic purposes. However, we will take for granted that these technologies provide a qualitative difference in the approach to medical services, justifying the PM label.

The ultimate ambition of PM is to prevent disease by understanding individuals’ health related risks, whether they are patients or healthy people. This can be done by profiling the potential for developing disease through the collection and analysis of large amounts of data from various sources. Current projects try to demonstrate how such strategies can be used in preventive measures within a framework of precision health (Schüssler-Fiorenza et al. 2019; Perkins et al. 2018). The opportunities here, for patients or non-patients alike, lie in the use of digital and computational technologies that enable us on the one hand to produce, collect and analyze big data in order to produce information, and on the other hand to interpret, give meaning and provide the potential to act on such information for individual benefit. An important part of this picture is using the potential of artificial intelligence—machine learning—to make sense of these huge amounts of data (Moore et al. 2019; Mesko 2017; Miller and Brown 2018).

Genomic medicine has already been adopted into medical practice, impacting fields like oncology, pharmacology, medical genetics and infectious disease (NIH 2019a, b), and has revolutionized the understanding and treatment of cancer (Berger and Mardis 2018; Sacha 2014; Liao et al. 2012). Examples from other areas include rapid, cost effective diagnosis of children with rare diseases and of very ill infants in neonatal intensive care units, based on the interpretations of the vast amounts of data from whole genome sequencing (Gyngell et al. 2019). Although a diagnosis is not equivalent to more treatment options and prolonged life, it is a step towards the improved health and well-being of these children. These and other cases contribute to current discussions concerning the use of genomics in screening programs in general and in newborn screening in particular (Powell 2018; UNCHealthCare 2019; Chowdhury et al. 2013; Brothers et al. 2019).

Right now there are several research initiatives that aim to prove that PM really can work. They explore to what extent so-called rich big data from individuals, including information from genomic sequencing, can predict an individual’s disease risk. The ultimate aim of PM is to extend people’s healthy life through improved prevention and early detection of, for example, cancer and age-related chronic disease (Perkins et al. 2018; Price et al. 2017; Schüssler-Fiorenza et al. 2019). Some of these strategies have positive results, indicating that with a sufficient amount of research participants providing a huge amount of data analyzed with novel artificial intelligence approaches, there is hope for the useful realization of the ideals of PM (Mesko 2017; Fogel and Kvedar 2018; Perkins et al. 2018; Williams et al. 2018). Our assumption is that the realization of these possibilities will have a significant impact on the relationship between medical professionals and the users of this information, and a key to study this impact is how it affects relations of trust.

## Trust and autonomy in medicine

Trust is a basic element in any medical intervention. People seek consultation or treatment in situations where they are vulnerable, and the procedures regularly involve actions that transgress the limits of privacy or personal space, physically or mentally. This requires that the patient *trusts* the person or institution. Trust is understood as an act where a person voluntarily leaves something of value to her in the power of somebody else. Trust concerns both the competence *and* good will of the trustee, where only the latter is descriptive of a moral relationship. In Jon Elster’s words, trust is “to lower one’s the guard” (Elster 2007, p. 344), making oneself vulnerable. Trust is a primary condition of social relationships in the sense that you have to trust in order to mistrust. As Annette Baier states: “We inhabit a climate of trust as we inhabit an atmosphere and notice it as we notice air, only when it becomes scarce and polluted” (Baier 1986, p. 234). Furthermore, trust in others expresses the assumption that they take responsibility for one’s interests.

There are two very different understandings of the phenomenon of trust, in medicine usually connected to the role of patient autonomy in clinical practice. The first one sees trust as fundamentally asymmetrical, with the parent–child relation as a paradigmatic example. This is what can be called non-cognitive trust, as trust on this account is not knowledge-based. Knud Løgstrup (1991) says that the human condition is from the start characterized by vulnerability and dependence on others, a point also central to the argument in Baier’s influential paper on trust (Baier 1986). Thus, trust is part of the basic condition of human life. This is not limited to the child’s situation. Many of our experiences as autonomous adults are also situations where we are dependent on the good intentions of others and most interactions in close relationships such as family relations and friendship have this kind of trust as an unquestioned basis. A man does not need to justify trust in his wife—given that they have a well-functioning marriage. On the contrary, if someone asks whether he trusts her—or why he trusts her—his most reasonable response would be to wonder whether they know something he is unaware of; something that he ought to know about her. Trust describes this kind of relation where we leave our life and interests in the hands of somebody else without considering when, and to what extent, they are trustworthy. Thus, it is fundamentally asymmetrical—it is not based on the trustor justifying the trust or controlling the trustee to do as promised or assumed. There is a moral dimension here. This blind or unconditioned trust is an expression of how one regards the other—and at the same time it contains an implicit challenge to the other of living up to this moral perception.

The classical doctor-patient relationship as described in academic literature (Campbell et al. 2001) belong to this category. It should be emphasized that the following account is an idealized typology, not an empirical description, although Tolstoy’s 1886 novella *The Death of Ivan Illyich* is presented as a paradigm of such relationships (Campbell et al. 2001). This typology of the patient prior to the autonomy turn sees her in a vulnerable position *vis-a-vis* health personnel who possesses the competence which the patients lack. Trusting them was the only option for alleviating the patient’s suffering. This also implies that the doctor carried the whole responsibility for the appropriateness of the intervention. The patient not only had to trust the doctor’s competence, but also his judgement. The professional role based on knowledge not available to non-experts gave an unquestionable authority, according to this typology. It is reasonable to call this blind trust, though it does not follow that it is an irrational act. If you have no alternatives to suffering, non-cognitive trust in authority is a rational choice.

Although patient trust in earlier times was more nuanced than allowed for on this account, it was accepted that doctors made decisions concerning the patient’s treatment without consulting the patient. Likewise, he could withhold information or even mislead the patient if that was considered beneficial for the patient’s healing. Being trustworthy meant making decisions based on authority, without involving the patient in the decision. This was not a trust based on knowledge, properly speaking, as the patient had little basis for questioning the doctor’s decisions. The only alternative was not trusting the doctor. In reality, the picture was more complex, for example people with resources and living in urban areas could choose one doctor over another based on knowledge of their respective therapeutic histories or their perceived trustworthiness, i.e. their ethos. Traditional folk medicine also provided alternatives to the authority of the medical profession. Still, compared to the present ideal of patient autonomy, the primary characteristic of the historical doctor-patients relationship is of non-cognitive trust.

This is in stark contrast to the arguably dominant approach to trust in mainstream political and moral philosophy, cognitive trust, which emphasizes the significance of knowledge and control. The paradigmatic example is the “encapsulated interest” account proposed by Hardin (2006). Such accounts of trust are based in a certain—more or less Hobbesian—anthropology of suspicion, treating trust as something that needs to be explained or justified. On this account, trusting without having experience and knowledge of the trustworthiness of the trustee is irrational. Trust is rational when we have reasons to think that the person or institution we trust will take care of our interests. Thus, trust is a matter of well-founded predictions about the future behaviour of those we interact with. The reason why we tend to trust those close to us more than others is that we know more about them and are better placed to predict how they will handle the values and interests we leave in their care. Blind trust of the type described initially is considered naive and immature. The paradigm of cognitive trust is a symmetrical voluntary relationship for mutual benefit.

The turn towards patient autonomy, expressed in the principle of informed consent, but also in an increasing tendency for patients to take charge by asking for second opinions, forming interest associations and actively searching for knowledge concerning their condition, has a long history with several recent drivers (Kilbride and Joffe 2018). The development of information technology in general and the creation and expansion of the internet in particular have been key factors (Diviani et al. 2019). Easy and affordable access to knowledge through scientific literature, popular versions thereof and the organization of patient interest groups worldwide have changed the potential for acquiring information relating to one’s own health and disease (Jacobs et al. 2017).

Taking this a step further, there are now even more personal, more intimate options of access to personal, health related data, such as data from direct-to-consumer genetic or even genomic testing (Hogarth and Saukko 2017; NIH 2019c). Recently, everyday use of data from usable, and to some extent reasonably priced, wearable personal sensors connected to a smart device via various apps has become mainstream in many cultures (Gambhir et al. 2018; Topol et al. 2015). These devices also demonstrate the increasingly blurred distinctions between technologies aimed at health promotion, managing everyday life and entertainment. Less common, but probably of increasing significance in health management, is the use of data from ingestible, subcutaneous or implantable sensors (Rich and Miah 2017; Cappon et al. 2017; Bigelow et al. 2016).

Such technologies have provided new sources of information on health status, diagnostics and treatment options, in many ways giving patients increased control over their own health. This turn has radically changed the trust dynamics of the doctor-patient relationship. Even if the patient wants to leave the decision to the doctor in non-cognitive, ‘blind’ trust, this is not an option. Autonomy is not only a right, it is also a duty, as expressed in informed consent requirements. In addition to demanding correct and understandable information, it is also required that the patient has read and understood the information. Thus, she must orient herself in the information and seek to get a grasp of it, while the health worker ensures that the information is understood. One could argue that today’s informed consent procedures is not grounded in a moral concern with patient empowerment, as expressed in the Helsinki Declaration, but is a risk management measure for the health services. Still, the demand on the patient for exercising autonomy is the same, and the patient has the final word in making the decision whether or not to carry out the proposed procedure.

The technological developments making information easily accessible enable the patient to second-guess medical personnel. The doctor becomes an equal—a discussion partner providing the patient with the required information for a sound decision. As we have seen, this is still a trust relationship, but a rational, reflexive kind of trust between equal partners. Although the doctor is a gatekeeper in virtue of having superior knowledge, the patient has the power to both act or not act on the knowledge obtained and hold the doctor accountable in case she fails to provide the right and best treatment. Although the ideal of non-directiveness is strong in these decision situations, it is clearly problematic both as a description of what is going on as well as indicating what ought to happen in these situations (Clarke 2017). The medical expert cannot be a trustworthy discussion partner without being engaged in the decision process.

This must be a matter of shared decision-making, implying that the patient must trust the doctor, but the basis for the trust is reflection on the information provided and the impression of the doctor’s competence and judgement. More importantly, the doctor is the one who is actually performing the treatment, and the patient is still leaving her health and well-being in the doctor’s hands. She is making herself vulnerable, but after carefully considering the options. Thus, the informed consent regime of modern medicine is making the patient’s consent a case of trust based on careful consideration of available information. A normative basis supporting this turn towards a new form of engaged trust is found in Kant’s moral philosophy.

### Kant on enlightenment, autonomy and trust

In the short tract *What is Enlightenment* from 1784, Immanuel Kant writes that “Enlightenment is man’s^1^ emergence from his self-imposed immaturity. Immaturity is the inability to use one’s understanding without guidance from another. … It is so convenient to be immature! If I have a book to have understanding in place of me, a spiritual adviser to have a conscience for me, a doctor to judge my diet for me, and so on, I need not make any efforts at all” (Kant 1983). Enlightenment is an ideal we ought to strive for, and in this context, becoming mature and taking responsibility is the same as exerting autonomy. This unwillingness to become autonomous is explained as stemming from “laziness and cowardice”. It is worth noticing in the context of trust in medicine and health services that Kant regards leaving decisions to the physician to be a case of immaturity, something we have a duty to abandon.

But this attitude of leaving your interests or purposes in the hands of what Kant calls guardians, i.e. some authority or expert, is what is usually called non-cognitive trust. In the standard trust literature this unconditional form is called ‘blind’ trust, and is typical of the kind of trust a child or a helpless person must exert. But it is also the kind of trust that you find in close family or friendship relations. As Pedersen (2012) points out, this is a passive trust which in this Kantian context can aptly be termed ‘lazy’ trust when the trustor is a competent adult. It is trust based on cognitive and moral laziness, not the result of an active, reflexive trust. The Kantian message on enlightenment is not that we should replace lazy immaturity with an anti-social self-sufficiency; the message of the tract on enlightenment is that we have a duty to go from lazy to active, reflexive trust.

Although it is easy to remain immature, the external obstacles to emancipation are still significant even if one does attempt to tear oneself out of this pleasant state, according to Kant. Authorities in different sectors, including the health services, want to remain guardians and strive to discourage and even prevent people from taking responsibility. “Thus, it is difficult for any individual man to work himself out of the immaturity that has all but become his nature. He has even become fond of this state and for the time being is actually incapable of using his own understanding, for no one has ever allowed him to attempt it” (Kant 1983). This authoritarian tendency is not necessarily caused by self-interest, it may well be due to a well-intentioned paternalism.

On Kant’s view, enlightenment is first and foremost a task for the whole public or society, and one that is only achieved slowly through history. Still, everyone has a duty to live up to the ideals of enlightenment, and Kant famously held that ‘ought implies can’. This is usually taken to mean that for something to be a duty, it is a presupposition that it be possible to carry out. It follows that, according to Kant, it must also be possible for the individual to reach the enlightenment ideals. It is evident from his examples of our enlightenment duties that this is a task we all have in everyday matters such as choice of diet, as well as when debating public issues. But then it is required that the individual is not prevented in the process of maturity by the authorities, and is also given the necessary tools. Public authorities must provide an empowering infrastructure. In order to achieve active trust, we must be given the cognitive tools for challenging authorities, as well as trustworthy counterparts. This implies that enlightenment understood as mature autonomy is not a task for the individual alone. It is a relational, intersubjective competence, based on a trust-building infrastructure.

Trust—or at least trustworthiness—is at the core of Kantian ethics, according to O’Neill (2002). Kantian autonomy is not merely self-determination, we must also be able to communicate our reasons to others, making them intelligible. This is what O’Neill calls principled autonomy, in contrast to John Stuart Mill’s individual autonomy, a reflexive choice under the Kantian idea of self-legislating reason. Arbitrary, irrational and harmful choices cannot be made intelligible in this sense. Deception and coercion are ruled out by the Categorical Imperative demands of universalizability and respect for humanity (O’Neill 2002). Although the primary trust-related duty derived from principled autonomy is trustworthiness, there are also moral implications on the trustor’s side of the relationship. Replacing lazy with active, reflexive trust, is not turning from naive faith to suspicious mistrust. O’Neill emphasizes, like Baier (1986), that it is impossible not to trust at all, a point also forcefully made by several social scientists discussing the contemporary risk society (Luhmann 2000; Beck 1992; Giddens 1990). In order to function in this society and for the society to function, we must trust people, social institutions and technologies, at least to some extent. However, this pragmatic point does not exhaust a Kantian approach to trust—there is also a duty to trust.

Respect for autonomy means expecting others to act morally. The second formulation of the Categorical Imperative states that we should act on a maxim treating every human being not merely as a means, but also as an end in themselves. Being an end in oneself is the same as being able to act according to the principle of self-legislation, avoiding deception and coercion. Another way of saying this is that treating someone as an end in themselves is treating them as trustworthy by actively trusting them. This is not lazy trust, but trust based on the principles of morality demanded of the ideal of enlightenment. By actively trusting someone we pose a moral challenge to them: “I trust you; show me that you are trustworthy.” This may seem an odd reversal, as one would think that trust should be earned through the trustee’s trustworthiness. But on Kant’s account, we do have an independent duty to trust, although we will see that this is not unconditional.^2^ It is also worth noting that similar accounts of ‘moralistic’ or ‘altruistic’ trust, which go beyond strategic and predictive understanding of the phenomenon, have been explored in contemporary social science literature (Uslaner 2002; Mansbridge 1999).

Even if we have a principled duty to trust, Kant has no illusions as to the inherent trustworthiness of humans, as is evident from the doctrine of radical innate evil, exemplified both in the ‘state of nature’ and among the most ‘civilised’ people. He says this evil is evident in the “secret falsity even in the closest friendship, so that a limit upon trust in the mutual confidences of even the best friends is reckoned a universal maxim of prudence in intercourse” (Kant 1960). There is a tension between the ideal demands of morality regarding trust and trustworthiness, and this prudential caution against unconditional trust. This tension is discussed in more detail elsewhere (Wood 1999; Pedersen 2012). The main point for our analysis is that resolving this tension in everyday morality is a task for the active reflexivity required of enlightenment, and makes regress to ‘lazy’ trust morally and prudentially unacceptable.

Patient autonomy—on this account—does not replace trust, but represents a turn from ‘lazy’ to active trust, where the patient issues a challenge to the physician for living up to the trust placed in her competence and good will. The patient has a duty to place active trust in the health personnel, taking part in deliberation and decision-making, respecting the competence of the doctor and expecting her good will, but always remaining open for questioning both aspects of the relationship. In order to do this, access to relevant information is required. But without the doctor in the dual role of taking responsibility for the quality and understandability of the information, it is of little value as a basis for reflexive trust. For a patient to undertake active trust it is necessary to take charge of their own situation, actively seeking and evaluating information and making decisions on this basis. But as Kant says, this is only possible if there are guardians that enable this empowerment by making relevant information available, and guaranteeing its relevance for the reflexive engagement. This account fits well with the anti-paternalistic autonomy turn in health care, though a further turn towards PM raises new problems of complexity, interpretation and expertise. This has led some central stakeholders to question the wisdom of the patient empowerment rhetoric and advocate a moderate return to paternalism.

### Personalized, stratified or precision medicine

Juengst et al. (2016) point out that there has been a rhetorical shift in recent years from ‘personalized’ to ‘precision’ in genomic medicine, and they argue that this is related to a shift in focus from the individual patient to groups, and also to an altered perception of responsibility:

The first is a turn away from “patient empowerment” and toward expert-mediated decision-making in the clinical setting…. The second is to broaden the movement’s focus from “individualizing” treatments for particular patients to using genomic profiling on behalf of the interests of extended families, minority groups, and national populations. (Juengst et al. 2016, p. 22)

One of the reasons is that the expected diagnosis and treatment will not be truly personal in the sense of being individual, but is a way to stratify patient groups to achieve more precise diagnosis for relevant subgroups. What is more important in our context, is that the change in name is connected to other shifts, namely “a renewed insistence on professional gatekeeping in the clinical application of genomic medicine and an increased interest in the public health uses of population-level conceptualization of genomic variation” (Juengst et al. 2016, p. 25). Actually, the authors think that there is little potential for patient empowerment in PM, given the way key actors present and talk about how one should deal with genomics information. The genomic information will be more suitable as a diagnostic tool for the expert, due to the sheer amount of information in need of interpretation and validation. We would add that PM depends on technology not only for the production of numerous data points but for the interpretation of these. This raises the general challenge of interpretation of “Big Data”. Someone, or rather a collaboration of multiple ‘someones,’ develop the algorithms which help experts and non-experts to make sense of the data. Thus, although not visible to the patient, there are several ‘experts’ in addition to medical personnel involved, such as bioinformatic experts, biocurators and artificial intelligence. And these collective someones, experts supported or replaced by technology, make decisions that affect the outcomes and make the possibilities for lay people alone to take control of the information even less likely. It is too complex. Thus the informants talk about “shared decision-making” in a model where professionals coach patients, as well as withhold what they regard to be unnecessary information (Juengst et al. 2016).

This is clearly a return to a form of mild paternalism that is contrary to the ideal of patient autonomy predominant in contemporary medical ethics. One may suspect that such paternalism is a reality in many clinical settings where patients may prefer to trust the judgement of the expert. Still, there is a significant distinction between a patient freely choosing to leave a decision to a doctor and a doctor deciding for the patient, because the doctor decides that is best for the patient. Now, the potential for a personalized medicine in the sense of an individualized as opposed to a stratified diagnostics and treatment may be a vision that will never materialize. But this does not mean that the development in genomics alters the decision situation in a way that justifies narrowing the room for enhanced patient control.

It is doubtful that it is morally or legally acceptable on any ethical approach that the physician decides to withhold information she finds irrelevant if the patients want access to this information. The Kantian account would certainly reject it. It is likewise unlikely that the physician can regain the gatekeeping position of coaching the patient choice unless this is a role given her by the patient, opting for ‘lazy’ trust. The respondents in the interviews cited in the Juengst article may wish to take that role and find this to be a more sustainable solution for handling what some have called the information tsunami of genomics, but the time for medical paternalism is over. Moreover, the public’s genetic literacy will increase, and technological tools will probably make the information much more accessible than envisioned in this article, giving patients further tools for guidance in making choices in addition to their physician. In short, there are ethical, juridical, social and technological reasons for rejecting a return to medical paternalism in a future PM, even though the term ‘empowerment’ is not the most appropriate for this enhanced potential for patient control. Despite this, we hold that the physician may have a key gatekeeping function in the new PM landscape.

This is the story of enlightenment, as it was understood by Kant, where maturity means taking responsibility. Maturity does not entail solipsistic decisions, but involves a real or virtual communicative intersubjectivity. There is one important factor in Kant’s account of enlightened maturity missing in this turn towards patient autonomy in medicine: the patient does not empower herself. The empowerment is more or less enforced by social, ideological and technological forces. In order to make PM as empowerment a reality, we also need to pay attention to this missing aspect. Autonomy cannot be imposed from the outside.

### The necessity of trust

O’Neill, as well as a number of influential sociologists, has argued that we need to trust for pragmatic reasons, namely to handle the complexities of modern risk societies. This is especially relevant for modern medicine. Medicine becomes more complex due to increased knowledge and improved, more advanced technologies. It enables better treatment for each individual case, greatly improving survival and recovery rates, but at the cost of simplicity and understandability. Here we do not primarily talk about asymmetrical or symmetrical trust, or of paternalism versus autonomy. We cannot say that this is a case of trusting some authority blindly because the situation is not one where we lack knowledge or understanding of something that an authority—a doctor or some other expert—knows or understands fully. Nor do we trust because we have experience with the medical services and can predict their behaviour from past actions or from insight into their motives, and we know that our interests are incorporated in theirs or some other typical account of cognitive trust.

On this account, we trust because we have no possibility to understand the technology and control whether it is beneficial to us, and have no alternatives to trust. This is especially clear when it comes to PM, based in genomics and other -omics technologies. The information is developed by the use of complex technologies, made possible by an interdisciplinary cooperation between a number of fields and techniques, where there is no single authority in charge. The knowledge is distributed and to a large extent de-personalized. There is a doctor to trust or distrust, but as she is not in control of this interdisciplinary technological complexity, apparently the trust is at least partly misplaced. Although there is no option but to trust, this trust does not encompass the whole field. Trust is the default attitude regarding health information, but the patient herself must take charge of the information, evaluate the sources and decide who, what, when and to what extent trust is warranted.

### Promises and perils of PM

We can sum up the promises and perils of PM as described in the first part as follows: We have an increased production of data points with an accompanying access to their possible interpretation—and increased reliability in the latter because of the former. This paves the way for increased tailoring of diagnostics, treatment and prevention. It will also enable increased participation and patient or user empowerment, and reduced expert power and medical paternalism. At the same time there is an increase in information ‘noise’. It is difficult to decide what information is relevant due to the sheer amount of data produced. These problems are increased by the interdisciplinary nature of genomics and other technologies essential for PM. No one has competence in everything, so the interpretation competence is distributed. The situation gets murkier due to the replacement of human expertise with technology. This gives, in principle, increased reliability of the data and improved access for all involved parties. On the other hand, these are non-accountable autonomous technologies developing according to trajectories only partly under human control. In addition, the source and logic of interpretations based on these technologies are partially hidden, preventing a cognitive basis for trust.

We thus have a better and more detailed but also more complicated picture, disturbed by noise. We have a transition from one or a limited number of experts to many, including non-human ones, with more uncertain accountability landscapes. We have increased opportunities for the individual with an accompanying increase in choices and required involvements. Although having choice is good, it does not follow that any increase in number of choices and options is good. The same can be said concerning involvement. Increased reliability is obviously good, but hidden interpretations and blurred accountability are not. This gives the individual patient or healthy user more autonomy, on condition that she develops higher personal competence in the relevant fields. This makes autonomy a requirement, a task, more than an opportunity or right. This is in line with the Kantian idea of principled autonomy, although for Kant this is not a necessity due to context, but a moral demand. Given this picture of PM, principled autonomy becomes a necessity, which makes it a contradiction in terms. Forced autonomy is no autonomy.

### Institutional conditions for PM trust

The autonomy turn in modern medicine makes a return to paternalism impossible. It is also morally wrong; we should not return to ‘lazy’ trust. The development of genomics medicine towards empowering PM strengthens this dual point. The only option adequate for the technological realities and acceptable in an enlightened modernity is active, reflexive trust. However, it is a fact that human beings have different cognitive capacities, giving us different possibilities for exercising this reflexive engagement. People will need help to interpret the data to varying degrees, with a similarly varied need for help when making informed choices. Thus, at least some will still need the advice of an expert with good knowledge in general medicine, genomics and other -omics technologies. But this expert must also have relevant experience. They will furthermore need directive guidance from an expert with similar good knowledge and experience. This is not trivial, but an essential, moral point.

Kant’s principle ‘ought implies can’ means that active, reflexive trust in PM presupposes that the trustor has sufficient knowledge and understanding to fulfil the requirement of taking charge of her own health. As expressed above, we can interpret Kant as stating that there must be a trustworthy infrastructure in place, enabling the individual to exercise their autonomy for the individual to live up to the obligation to become enlightened. The authorities must create a system for making available relevant information enabling adequate deliberation. The existence of a trustworthy counterpart is equally important. There can be no trust without a trustee, that is, someone who is accountable. But how is that possible, when the single physician is replaced with an opaque collection of experts and technologies? First of all, this means that PM trust requires a system of institutional controls of information and diagnostics. This must function according to the principle of institutionalized distrust (Luhmann 2000). When we enter an airplane or some other public transport system, or we give our personal information to some public authority, we know there are systems for checking that these systems are functioning properly. The best way to ensure this is systematically looking for flaws, which is the same as using an approach of systematic distrust. Although we do not know the specifics of these institutional control systems, we understand how they work, since this is a standard approach to risk management in the modern technological society. This means that trust based on institutionalized systematic distrust is a kind of reflexive, not blind, trust.

However, institutional distrust is not sufficient for active reflexive trust, at least not when the issue at stake is our own health. It must be supplemented with a system of human accountability for the use and understanding of technology-generated data. This means that the physician or similar medical expert is crucial for a functioning PM. Although there are several different experts and technologies involved in the practices gathered under the PM umbrella, the person depending on these different instances of expertise needs to trust the system as such. This means that she needs a contact point for at least three different purposes. First, she needs someone to explain and discuss the medical advice and choices made available by the PM system. Second, she needs someone to direct her to the right expertise when she needs more precise and detailed background information, in order to take charge of her own medical information. Third, she needs someone to be accountable for the correctness of the information and soundness of the advice. Even if the Kantian ideal of active reflexive trust demands that the patient takes responsibility for her own fate, this cannot remove the demands of trustworthiness and accountability of the medical expertise. Expert accountability is a precondition for active trust. Even if we may speak of trust in institutions and systems, the communication required for reflexive trust needs a focal point.

Only a competent person with good will can take this role as focal point for the trust in the PM system of experts and technologies. However, this is not a return of the paternalist gatekeeper, as suggested by some of the professionals interviewed by Juengst and colleagues. Even if there is an asymmetry in the knowledge and accountability between the patient and the medical expert, the communication, reflection and decision based on the information provided must be shared on an equal footing. There is no room for paternalism in this relationship, presupposing the Kantian moral ideal of the enlightened patient. Given this ideal, we suggest that the medical expert should assume the role of a true friend in Kant’s trust analysis, “participating and sharing in the other’s well-being through the morally good will that unites them” (Kant 1996, p. 215). Here the medical expertise plays a key role in ensuring a sound basis for active trust in PM. We need medical personnel with competence in genomics as the contact point between the individual and the complex of experts and digital and other technologies underlying PM. Only a human being capable of autonomy can be worthy of active reflexive trust, because trustworthiness implies accountability.

As we have stated above, this account of trust in PM presupposes an ideal of the empowered, active and reflexive patient; something we should strive for but which is seldom realized in the everyday clinical situation. However, even if the patient fails to live up to this ideal, the health personnel and other involved experts should act as if the patients assumed this empowered role. If they fail to expect autonomy and to provide room for the patient to take charge of her own treatment, their role will be that of the guardians described by Kant: “[T]hese guardians make their domestic cattle stupid and carefully prevent the docile creatures from taking a single step without the leading-strings to which they have fastened them” (Kant 1983). Treating someone as autonomous opens the path for them to develop autonomy. If the patient fails in her duty of enlightenment and asks the health personnel to take charge of the situation, analyze the data and decide on a course of action, this is still closer to empowerment than a lazy trust in a paternalist expert.

## Conclusion

We have discussed how the promises of personalized medicine present an extension of the emphasis on the empowerment of the individual through active participation in managing her health, disease and wellbeing, characteristic of modern medicine. Personalized medicine is characterised by increased production of and access to data points, with increased reliability of the interpretations and accompanying tailoring of diagnostics, treatment and prevention to the individual. The ideal is to increase wellness by minimizing the layer of interpretation and translation between relevant health information and the patient or user. This promises a new level of autonomy through increased participation in treatment and prevention, and through that, increased empowerment of the individual.

This picture of the empowered individual taking responsibility for her own health information and treatment echoes Kant’s analysis of enlightenment as one’s emergence from a “self-imposed immaturity”. This is intrinsically connected to trust, which is at the core of Kantian ethics, with a moral demand for being trustworthy as well as a conditional duty to trust others. This is not straightforward in the envisioned PM landscape, where there are no obvious trustworthy counterparts. Realizing the promises requires a trust-fostering system of institutional controls of information and diagnostics, accessible for non-specialists, supported by medical expertise that can function as the accountable gatekeeper taking moral responsibility required for an active, reflexive trust.

## Footnotes

1 Kant writes “des Menschens,” which literaly means the gender-neutral “the human being’s”, indicating that enlightenment is not a task for men only.

2 One could ask why lazy trust is a violation of the second formulation of the Categorical Imperative, as it is not obvious that the physician is treated as a mere means in this situation. This is because it is not the physician that is denied being an end in themself, but the patient herself. Failing to exercise autonomy is treating humanity in one’s own person as a mere means—which is a crucial point expressed in *What is Enlightenment?*
